# Supplementary material for: Scoping review protocol: The chrononutrition factors in association with glycemic outcomes in adult population
Source: PLoS One. 2025 Feb 14;20(2):e0313931. doi: 10.1371/journal.pone.0313931 (PMC11828428; doi:10.1371/journal.pone.0313931)
Supplement: Attachment — Submitted filename: 1. Reply to Reviewers PLOS ONE.pdf [file pone.0313931.s002.pdf]

Dear reviewers from PLOS One,

We sincerely thank the reviewers for taking the time to review our manuscript and providing constructive feedback to improve our manuscript. We sincerely hope that you may reconsider our manuscript entitled "**Scoping review protocol: The chrononutrition factors in association with glycemic outcomes in the adult population**." **(Ref No: PONE-D-23-29076)** for publication in PLOS One. Enclosed is the revised version of our manuscript.

Below are our responses to the comments and recommendations.

Reviewers' comments:

Reviewer's Responses to Questions
Comments to the Author

1. Does the manuscript provide a valid rationale for the proposed study, with clearly identified and justified research questions?

The research question outlined is expected to address a valid academic problem or topic and contribute to the base of knowledge in the field.
Reviewer #1: Yes
Reviewer #2: Yes

Author's response: Thank you for your feedback.

---

2. Is the protocol technically sound and planned in a manner that will lead to a meaningful outcome and allow testing the stated hypotheses?

The manuscript should describe the methods in sufficient detail to prevent undisclosed flexibility in the experimental procedure or analysis pipeline, including sufficient outcome-neutral conditions (e.g. necessary controls, absence of floor or ceiling effects) to test the proposed hypotheses and a statistical power analysis where applicable. As there may be aspects of the methodology and analysis which can only be refined once the work is undertaken, authors should outline potential assumptions and explicitly describe what aspects of the proposed analyses, if any, are exploratory.
Reviewer #1: Yes
Reviewer #2: Partly

Author's response: Thank you for your feedback. We will address this point in detail within the specific feedback (point L) provided by the reviewers below.

---

3. Is the methodology feasible and described in sufficient detail to allow the work to be replicable?

Descriptions of methods and materials in the protocol should be reported in sufficient detail for another researcher to reproduce all experiments and analyses. The protocol should describe the appropriate controls, sample size calculations, and replication needed to ensure that the data are robust and reproducible.
Reviewer #1: Yes

Reviewer #2: Yes

Author's response: Thank you for your feedback.

---

4. Have the authors described where all data underlying the findings will be made available when the study is complete?

The PLOS Data policy requires authors to make all data underlying the findings described in their manuscript fully available without restriction, with rare exception, at the time of publication. The data should be provided as part of the manuscript or its supporting information, or deposited to a public repository. For example, in addition to summary statistics, the data points behind means, medians and variance measures should be available. If there are restrictions on publicly sharing data—e.g. participant privacy or use of data from a third party—those must be specified.
Reviewer #1: No
Reviewer #2: Yes

Author's response: Thank you for your feedback. We will address this point in detail within the specific feedback (point K) provided by the reviewers below.

---

5. Is the manuscript presented in an intelligible fashion and written in standard English?

PLOS ONE does not copyedit accepted manuscripts, so the language in submitted articles must be clear, correct, and unambiguous. Any typographical or grammatical errors should be corrected at revision, so please note any specific errors here.
Reviewer #1: Yes
Reviewer #2: Yes

Author's response: Thank you for your feedback.

---

6. Review Comments to the Author

Please use the space provided to explain your answers to the questions above and, if applicable, provide comments about issues authors must address before this protocol can be accepted for publication. You may also include additional comments for the author, including concerns about research or publication ethics.

You may also provide optional suggestions and comments to authors that they might find helpful in planning their study.

(Please upload your review as an attachment if it exceeds 20,000 characters)

Author's response: Thank you for your feedback.

Reviewer #1: The scoping review protocol is an interesting beginning for a research study. I have many minor modifications and few questions about the manuscript. The overall work look interesting and it could provide a good paper to fill the clinical evidence summarizing the information on chrononutrition factors in association with glycemic outcomes in adult population.

a) The introduction (both in abstract and the manuscript) should be enlarged in order to provide larger detail and context of the topic. Actually, is pretty limited. Why you haven't included grey literature in the studies selected? Please justify it.

Author's response: Thank you for your feedback. We appreciated your suggestion to expand the introduction. We have revised both abstract and manuscript to provided more detailed context and background on the topic the grey literature in the manuscript.

Abstract, Page 2, Lines 28 to 31: Chrononutrition, which examines the relationship between circadian rhythms and nutrition, has been associated with glycemic outcomes in adults. However, published data on delayed meal timing, increased meal frequency and frequent breakfast skipping have shown inconsistent glycemic outcomes due to variations in methodologies and populations studied.

Introduction, Page 3, Line 54 to Page 5, Lines 119: Diabetes is a lifelong metabolic disease with a rising global prevalence, projected to affect 260.2 million adults in the Western Pacific region by 2045 [1,2]. Despite the American Diabetes Association's lifestyle recommendations have suggested healthy diet and physical activity to regulate blood glucose levels, but current guidelines may be insufficient for effective diabetes management [3]. Emerging evidence increasingly highlights the critical role of circadian clock in metabolic regulation, introducing chrononutrition as a significant factor in diabetes management [4–6]. Recently, modern adults often engage in irregular eating patterns, including skipping breakfast [7,8], extending daily eating windows [9], reducing nocturnal fasting periods [10], and frequent nighttime snacking [11]. These behaviors are closely related to insulin resistance, leading to elevated glycated hemoglobin levels (HbA1c), increased postprandial and fasting plasma glucose levels, and a higher risk of diabetes [12–16].

Chrononutrition's key components, including energy distribution, meal regularity and frequency of meals, have been shown to influence glycemic outcomes [17]. Current evidence had extended its components to meal timing, specifically night eating [18]. Large-scale studies have demonstrated the association between eating later in the day and poor glycemic control [15,19]. For example, the National Health and Nutrition Examination Survey (NHANES) in the United State (US) found a significant association between the last meal time before bed and elevated HbA1c levels[15]. However, findings from the Korea National Health and Nutrition Examination (KNHANES) did not show a significant association between evening eating, defined as consuming more than 40% of daily energy between 6.00pm to 9.00pm, and adverse glycemic outcomes [19]. In contrast, a US study suggested that restricting eating to window from 4.00 am to 4.00 pm, with light dinner and energy distribution focused on breakfast and lunch could stabilize glucose levels and reduce fluctuations [20]. This statement is also in agreement with Asian studies, which indicated that hyperglycemia is associated with eating meals after 8.00pm [21,22]. Studies also indicated that fasting gaps allocated before sleep by refraining from late-

night dinner eating may regulate blood glucose effectively [23,24]. However, identifying specific time-related dietary factors that contribute to glucose intolerance remains complex, influenced by seasonal variations, daylight exposure and individual lifestyle factors [25–27].

Given meal timing is related to meal frequency, night fasting duration, and energy distribution of a day, this study explores their associations with glycemic outcomes. Previous studies have reported that irregular meals, specifically eating more than four meals or fewer than two meals daily, are associated with an increased risk of diabetes [28–31]. However, other studies have found no significant association between meal frequency and diabetes risk [10,32]. These discrepancies are likely due to variations in meal timing and frequencies, leading to inconsistent glycemic outcomes [29,33]. A systematic review suggests that restricting eating to 2 to 3 meals daily, within a feeding window of less than 10 hours and consuming the last meal 3 to 4 hours before sleep, may effectively manage glucose levels in individuals with type 2 diabetes [34]. However, this strategy's applicability to other populations, including healthy individuals [35], those who are overweight or obese [36], where the effects of meal timing remain uncertain. Furthermore, breakfast skippers delayed their first meal and reserved majority of their energy intake at evening time has provided mixed results on glycemic outcomes [37,38]. Thus, the available evidence showed inconsistency in the association between chrononutrition factors and glycemic outcomes.

Conventional methods for diagnosing glycemic outcomes, such as random blood glucose, fasting plasma glucose, the 75g oral glucose tolerance test and HbA1c levels may not reliably capture advanced interday or intraday glucose fluctuations [39]. Therefore, this study incorporates studies that using the ambulatory glucose profiles derived from continuous glucose monitoring sensors could provide a more advanced and comprehensive understanding of 24-hour glucose variability in individuals with hyperglycemia [40]. Understanding how chrononutrition factors interact and influence glycemic outcomes is essential, although health effects of combining different chrononutrition factors remain uncertain. A comprehensive evaluation of these factors could provide valuable insights into their association with glycemic outcomes. With the ultimate aim to improve the diabetes management, the present review will identify and analyzes the knowledge gaps regarding the association between chrononutrition factors and glycemic outcomes in adult population. Specifically, this review aims to:

1. investigate the association between chrononutrition factors (meal regularity, meal timing and meal frequency) and glycemic outcomes in terms of glucose indices, insulin indices and incidence of diabetes among adults.
2. describe the chrononutrition factors (meal regularity, meal timing and meal frequency) applied in various populations and their effect on glycemic outcomes (glucose indices, insulin indices and incidence of diabetes) of the populations.
3. examine the opportunities to integrate chrononutrition into medical nutrition therapy to prevent and control diabetes mellitus.

b) Why only studies from 2012? Please justify it.

Author's response: Thank you for your feedback. We intended to select studies from past ten years to capture the most recent and relevant research, reflecting the developments in this rapidly evolving field up to the time of our manuscript preparation and submission in 2024. To ensure our review includes the most up-to-date evidence,

we have now revised and extended our search to include studies published from 2014 up to 2024.

Abstract, Page 2, Lines 36 to 38: This review focuses on original articles published from January 2014 to 2024, involving participants aged 18 years and older, published in English, and encompassing experimental and observational studies.

Materials and methods, Step 2, Page 8, Lines 184 to 187: Studies that focus on: (1) adult population aged 18 to 60 years; (2) original journal articles published from 2014 to 2024; (3) the full text available in English will be included and (4) the experimental and observational studies.

c) In the introduction you report that in 2045 there will be 783 millions of people with diabetes. I could suggest also to introduce the actual incidence and prevalence worldwide or in some specific countries (such as US, Europe).

Author's response: Thank you for your feedback. We have included the projected incidence and prevalence of diabetes for the Western Pacific region in 2045 in the content.

Introduction, Page 3, Lines 54 to 55: Diabetes is a lifelong metabolic disease with a rising global prevalence, projected to affect 260.2 million adults in the Western Pacific region by 2045.

d) When you state that "In recent years, a growing body of evidence suggests that the circadian clock interconnects with nutrients, and impacts on body's physiological process, namely chrononutrition [7]", I expect at least several manuscripts and some systematic literature reviews that state this, not just a study.

Author's response: Thank you for your feedback. We have included several literature reviews for this statement.

Introduction, Page 3, Lines 58 to 60: Emerging evidence increasingly highlights the critical role of circadian clock in metabolic regulation, introducing chrononutrition as a significant factor in diabetes management [4–6].

e) In the objective of the scoping review you state that you will provide a definition of chrononutrition characteristics. What will you use for it? Maybe a concept analysis? Please provide larger detail.

Author's response: Thank you for your feedback. After further discussion with the team, we have decided to exclude the objective of defining chrononutrition characteristics. Instead, we will focus on our primary aim of investigating the association between chrononutrition factors and glycemic outcomes.

Introduction, Page 5, Line 111 to Page 6, Line 119: Specifically, the review aims to:
1. investigate the association between chrononutrition factors and glycemic outcomes among adults.

2. describe the chrononutrition factors applied in various populations and their effect on glycemic outcomes of the populations.
3. examine the opportunities to integrate chrononutrition into medical nutrition therapy to prevent and control diabetes mellitus.

f) Can you provide in the supplementary material the data extraction form that you will use in the scoping review?

Author's response: Thank you for your feedback. We have included the data extraction form in the Supplementary Material 2.

g) Can you provide larger details of the benefits of this review for government and policymakers?

Author's response: Thank you for your feedback. We have provided the details of the benefits of this review for government and policymakers.

Discussion, Page 17, Line 320 to Page 18, Line 334: At population level, the findings of the review are crucial for governments and policymakers. Incorporating circadian eating into clinical practice guideline, dietary recommendations and public health policies aimed at managing glycemic control and preventing diabetes could have significant benefits. Specifically, these guidelines might advocate for consistent daily eating start and end times, ensuring the last meal occurs before a certain clock time or within a defined period before bedtime. By identifying the most impactful chrononutrition factors influencing glycemic outcomes, resources can be efficiently directed towards targeted programs and initiatives, thus maximizing the return on investment in public health. Furthermore, this review has also highlighted the gaps in current research, promoting collaboration between government bodies, academic institutions, and healthcare providers. This cooperation is essential for driving innovation in chrononutrition and metabolic health research. The insights gained can also facilitate international collaborations, ensuring the country remains at the forefront of advancements in nutrition and public health. By developing of long-term strategies to reduce the burden of metabolic diseases, the review's findings can ultimately enhance the quality of life for the population.

h) Why using POE and not PICOTS? Please justify.

Author's response: Thank you for your feedback. We have chosen the PEO framework for this scoping review because it aligns more closely with our focus on the adult population, the exposure of interest, and the examination of glycemic outcomes. The PEO framework allows us to concentrate specifically on these aspects, whereas the PICOTS framework typically used for topics involving an intervention. It includes additional components such as time, setting, and study design, is more suitable for systematic reviews and meta-analyses where such details are essential. Our primary aim is to explore the association between chrononutrition factors and glycemic outcomes, making the PEO framework the most appropriate choice for our review.

i) When will you start the Scoping review?

Author's response: Thank you for your feedback. We plan to commence this scoping review in September 2024.

j) Please state also in the limitation of the study the fact that you are providing a Scoping review and not a Systematic Literature Review that will be more appropriate.

Author's response: Thank you for your feedback. We will include in the limitations section of the study that we are conducting a scoping review rather than a systematic literature review, acknowledging that the latter would be more appropriate for certain aspects of the research.

Limitation, Page 18, Lines 340 to 342: Thirdly, scoping reviews inherently provide broader but less in-depth information on a topic and serve primarily as a hypothesis-generating approach.

k) In addition, how will you compare the numerical outcomes? Please state it in the manuscript.

Author's response: Thank you for your feedback. We have revised the manuscript to clarify how numerical outcomes will be compared.

Materials and Methods, Step 5, Page 16, Lines 270 to 275: Descriptive statistics will be used to summarize the numerical outcomes across studies, providing an overview of the findings related to association between chrononutrition factors and glycemic outcomes. The authors will explore trends and patterns in the data to identify consistent findings or disparities among the studies. This approach will enable authors to compare and contrast the results, offering a comprehensive understanding of the association between chrononutrition and glycemic outcomes.

Reviewer #2: I am thankful to the authors for giving updated information on The Chrono nutrition factors in association with glycemic outcomes in the adult population. The different parts of this review manuscript is well prepared. This review article can be moved further after fulfilling the necessary suggestions which are listed below.

Major comments

I) The discussion is too narrow. The discussion parts fail to meet the standard outcome of this review

Author's response: Thank you for your feedback. We acknowledge that the discussion section needed further expansion to meet the expected outcomes of this review. In response, we have revised the discussion to provide a broader and more comprehensive analysis of the findings.

Discussion, Page 17, Lines 300 to 334: This scoping review will utilize the transparent and reproducible procedure throughout the review process. The rigorous selection of data sources, search strategy, data extraction, and the quality assessment methods will ensure broad coverage of the existing literature. An assessment of study quality, while not mandatory in scoping reviews, is a strength of this review as it helps identify potential biases in the study findings. Additionally, this quality assessment will serve as a precursor to a full systematic review and meta-analysis. Current evidence on the association between chrononutrition-related factors and glucose outcomes remains inconclusive [17,53–56]. This review aims to elucidate the optimal meal timing, meal frequency, night fasting duration and other chrononutrition factors that can optimize glucose levels and enhance glucose responses.

Addressing the existing scientific gaps, the synthesized evidence will provide valuable guidance for supporting, developing, and implementing effective lifestyle interventions[9] . At the individual level, tailoring food intake timing to align with an individual's biological clock, chronotype and circadian rhythm through personalized chrononutrition interventions could be a feasible and cost-effective approach for regulating abnormal glycemic outcomes [57]. Moreover, integrating chrononutrition interventions with strategies to address food insecurity is essential to ensure consistent meal timing. Combining chrononutrition with other lifestyle interventions, such as those focused on enhancing diet quality or promoting sleep health, could further optimize the health benefits of these approaches [6].

At population level, the findings of the review are crucial for governments and policymakers. Incorporating circadian eating into clinical practice guideline, dietary recommendations and public health policies aimed at managing glycemic control and preventing diabetes could have significant benefits. Specifically, these guidelines might advocate for consistent daily eating start and end times, ensuring the last meal occurs before a certain clock time or within a defined period before bedtime. By identifying the most impactful chrononutrition factors influencing glycemic outcomes, resources can be efficiently directed towards targeted programs and initiatives, thus maximizing the return on investment in public health. Furthermore, this review can highlight gaps in current research, promoting collaboration between government bodies, academic institutions and healthcare providers. This cooperation is essential for driving innovation in chrononutrition and metabolic health research. The insights gained can also facilitate international collaborations, ensuring the country remains at

the forefront of advancements in nutrition and public health. By developing of long-term strategies to reduce the burden of metabolic diseases, the review's findings can ultimately enhance the quality of life for the population.

m) The level of English must be improved.

Author's response: Thank you for your feedback. We acknowledge the need to enhance the level of English in the manuscript. We have revised the text to improve clarity, coherence, and overall language quality.

n) In the abstract section, the authors cannot justify their argument. It should be more precise about the core findings (should include scientific outcomes)

Author's response: Thank you for your feedback. We appreciate your suggestion and have revised the abstract to include a more precise justification and a clear summary of the core scientific findings. This adjustment ensures that our key outcomes are effectively communicated.

Abstract, Page 2, Lines 28-31: Chrononutrition, which examines the relationship between circadian rhythms and nutrition, has been associated with glycemic outcomes in adults. However, published data on delayed meal timing, increased meal frequency and frequent breakfast skipping have shown inconsistent glycemic outcomes due to variations in methodologies and populations studied.

Minor comments

o) L117, more clearly the objective by adding one or more sentences and also emphasizes using review.

Author's response: Thank you for your feedback. We have revised the manuscript to provide a clearer and more detailed explanation of the review's objectives.

Page 4, Line 108 to Page 5, Line 119: With the ultimate aim to improve the diabetes management, the present review will identify and analyzes the knowledge gaps regarding the association between chrononutrition factors and glycemic outcomes in adult population. Specifically, this review aims to:
1. investigate the association between chrononutrition factors (meal regularity, meal timing and meal frequency) and glycemic outcomes in terms of glucose indices, insulin indices and incidence of diabetes among adults.
2. describe the chrononutrition factors (meal regularity, meal timing and meal frequency) applied in various populations and their effect on glycemic outcomes (glucose indices, insulin indices and incidence of diabetes) of the populations.
3. examine the opportunities to integrate chrononutrition into medical nutrition therapy to prevent and control diabetes mellitus.

p) L127, 9.00pm to 9.00 pm or 21:00.

Author's response: Thank you for your feedback. We have revised the manuscript for clarity and consistency in time notation.

Introduction, Page 3, Lines 72 to 74: However, findings from the Korea National Health and Nutrition Examination (KNHANES) did not show a significant association between evening eating, defined as consuming more than 40% of daily energy between 6.00pm to 9.00pm, and adverse glycemic outcomes [19].

q) L147, Materials and methods. The authors are encouraged to add a flowsheet at the end of describing steps (steps 1-5).

Author's response: Thank you for your feedback. We have added a flowchart at the beginning of describing steps.

Materials and Methods, Page 5, Lines 124 to 124: Specifically, five steps of methodology will be adopted in our scoping review in Figure 1.

Figure 1: The figure illustrates the steps involving in the scoping review methodology.

r) pL214-215, Given the initial searches yielded 627 records…….. In which criteria did authors find those 627 records? Keywords? Which keywords?

Author's response: Thank you for your feedback. The initial search in July 2022 yielded 627 records (total from A29 AND B21) based on the all keywords specified in our search strategy (Table 1) due to the time elapsed before the submission of this protocol, we have updated our search strategy in July 2024 to ensure that we capture the most recent and relevant studies based on the inclusion and exclusion criteria. The revised search will be reflected in the updated records and analysis.

Materials and Methods, Step 3, Page 12, Lines 205 to 207: Given the initial searches yielded 491 records, the title and abstract will be screened according to the eligibility criteria outlined above.

Table 2: The complete searching strategy and pilot searches in several electronic databases.

s) L306-312, it is better to make another paragraph stating the possible conclusion of this review.

Author's response: Thank you for your feedback. We have revised the manuscript to address your suggestion. The conclusion has been separated into its own paragraph to clearly highlight the anticipated outcomes and implications of the scoping review.

Limitation, Page 18, Line 349 to Page 19, Line 355: The authors anticipate that the scoping review will elucidate the association between delayed meal timing, frequent meal intake and increased breakfast skipping with glycemic outcomes. By exploring these chrononutrition factors in relation to glycemic control, the review provides valuable insights for the public health sector. This evidence can serve as basis for developing hypotheses that involve the development of evidence-based lifestyle intervention programs and support the integration of chrononutrition principles, ultimately aiding in the prevention and management of metabolic diseases.

7. PLOS authors have the option to publish the peer review history of their article (what does this mean?). If published, this will include your full peer review and any attached files.

If you choose “no”, your identity will remain anonymous but your review may still be made public.

Do you want your identity to be public for this peer review? For information about this choice, including consent withdrawal, please see our Privacy Policy.
Reviewer #1: No
Reviewer #2: No

Author’s response: Thank you for your feedback.
