# Supplementary material for: Scoping review protocol: The chrononutrition factors in association with glycemic outcomes in adult population
Source: PLoS One. 2025 Feb 14;20(2):e0313931. doi: 10.1371/journal.pone.0313931 (PMC11828428; doi:10.1371/journal.pone.0313931)
Supplement: Attachment — Submitted filename: 1. Reply to Reviewers PLOS ONE_02.docx [file pone.0313931.s003.docx]

Dear reviewers from PLOS One,

We sincerely thank the reviewers for taking the time to review our manuscript and providing constructive feedback to improve our manuscript. We sincerely hope that you may reconsider our manuscript entitled “**Scoping review protocol: The chrononutrition factors in association with glycemic outcomes in the adult population**.” **(Ref No: PONE-D-23-29076R2)** for publication in PLOS One. Enclosed is the revised version of our manuscript.

Below are our responses to the comments and recommendations.

**Reviewers' comments:**

1. Does the manuscript provide a valid rationale for the proposed study, with clearly identified and justified research questions?

The research question outlined is expected to address a valid academic problem or topic and contribute to the base of knowledge in the field.

Reviewer #1: Yes

Reviewer #2: Yes

**Author’s response:** Thank you for your feedback.

2. Is the protocol technically sound and planned in a manner that will lead to a meaningful outcome and allow testing the stated hypotheses?

The manuscript should describe the methods in sufficient detail to prevent undisclosed flexibility in the experimental procedure or analysis pipeline, including sufficient outcome-neutral conditions (e.g. necessary controls, absence of floor or ceiling effects) to test the proposed hypotheses and a statistical power analysis where applicable. As there may be aspects of the methodology and analysis which can only be refined once the work is undertaken, authors should outline potential assumptions and explicitly describe what aspects of the proposed analyses, if any, are exploratory.

Reviewer #1: Yes

Reviewer #2: Yes

**Author’s response:** Thank you for your feedback.

3. Is the methodology feasible and described in sufficient detail to allow the work to be replicable?


Descriptions of methods and materials in the protocol should be reported in sufficient detail for another researcher to reproduce all experiments and analyses. The protocol should describe the appropriate controls, sample size calculations, and replication needed to ensure that the data are robust and reproducible.

Reviewer #1: Yes

Reviewer #2: Yes

**Author’s response:** Thank you for your feedback.

4. Have the authors described where all data underlying the findings will be made available when the study is complete?

The [PLOS Data policy](https://c05y1x9s.r.us-east-2.awstrack.me/L0/https:%2F%2Fjournals.plos.org%2Fplosone%2Fs%2Fmaterials-and-software-sharing/1/010f01926da3ee31-e1098fc4-9bf6-478d-ac73-f4e42a9e89c3-000000/NpcVfNJRZ2OjOC5P2m5jtkkB32Q=179) requires authors to make all data underlying the findings described in their manuscript fully available without restriction, with rare exception, at the time of publication. The data should be provided as part of the manuscript or its supporting information, or deposited to a public repository. For example, in addition to summary statistics, the data points behind means, medians and variance measures should be available. If there are restrictions on publicly sharing data—e.g. participant privacy or use of data from a third party—those must be specified.

Reviewer #1: Yes

Reviewer #2: Yes

**Author’s response:** Thank you for your feedback.

5. Is the manuscript presented in an intelligible fashion and written in standard English?

PLOS ONE does not copyedit accepted manuscripts, so the language in submitted articles must be clear, correct, and unambiguous. Any typographical or grammatical errors should be corrected at revision, so please note any specific errors here.

Reviewer #1: Yes

Reviewer #2: Yes

**Author’s response:** Thank you for your feedback.

6. Review Comments to the Author

Please use the space provided to explain your answers to the questions above and, if applicable, provide comments about issues authors must address before this protocol can be accepted for publication. You may also include additional comments for the author, including concerns about research or publication ethics.

You may also provide optional suggestions and comments to authors that they might find helpful in planning their study.

(Please upload your review as an attachment if it exceeds 20,000 characters)

**Reviewer #1:** I have very few questions:

I think that your review should address all the gaps in the literature and trying to harmonize3 the outcomes and trying to investigate in deep in possible issues derived by discording results. You should provide a larger set of review objectives.
Why do you consider people within 18-60 years? Please justify it.

**Author’s response:** Thank you for your valuable feedback. We consider people within 18 to 60 years as adult age range because according to World Health Organization, individuals aged 18 years and older are considered adults in most contexts (WHO, 2015). The age range is important for ensuring that participants are legally capable of providing informed consent and are generally in a period of relatively stable physical and physiological functioning (WHO, 2015). Studies on adults beyond the age of 60 are most likely to be influenced by natural aging processes, such as changes in metabolism, body composition and increased prevalence of chronic diseases can create significant variations in health outcomes (Alan, 2023;Li et al. 2018). Additionally, the working age group (18-60 years) typically represents adults who are actively engaged in employment and have consistent lifestyle patterns related to diet, physical activity and sleep (WHO, 2015; Rudnicka et al. 2020). In line with your suggestion, we also recognize the importance of addressing gaps in the literature by harmonizing findings from discordant results. Our choice of this age range allows us to focus on a more homogenous population, minimizing confounding factors, and thereby improving the precision of our findings. Furthermore, the exclusion of participants over 60 ensure our review objectives remain focused on identifying patterns that are less likely to be influenced by aging-related variations, while also contributing to the larger body of literature that explores health outcomes in this population.

Citation:

a) Li Y, Zhao L, Yu D, Wang Z, Ding G (2018) Metabolic syndrome prevalence and its risk factors among adults in China: A nationally representative cross-sectional study. PLOS ONE 13(6): e0199293. <https://doi.org/10.1371/journal.pone.0199293>.
b) Gutterman, Alan, World Health Organization and Older Persons (January 1, 2023). Available at SSRN: <https://ssrn.com/abstract=4320044> or [http://dx.doi.org/10.2139/ssrn.4320044](https://dx.doi.org/10.2139/ssrn.4320044)
c) World Health Organization. (‎2015)‎. World report on ageing and health. World Health Organization. <https://iris.who.int/handle/10665/186463>
d) Rudnicka E, Napierała P, Podfigurna A, Męczekalski B, Smolarczyk R, Grymowicz M. The World Health Organization (WHO) approach to healthy ageing. Maturitas. 2020 Sep;139:6-11. doi: 10.1016/j.maturitas.2020.05.018. Epub 2020 May 26. PMID: 32747042; PMCID: PMC7250103.

**Pages 6 to 7, lines 141 to 145:** We considered individuals aged 18 to 60 years in this group represents a relatively stable period of physical and physiological functioning, minimizing the influence of aging-related factors that could complicate data interpretation. This age group also aligns with the working-age population, which typically has consistent lifestyle patterns related to diet, physical activity and sleep.

**Reviewer #2:** The manuscript title "Scoping review protocol: The chrononutrition factors in association with glycemic outcomes in adult population"" is well revised. The authors fulfill each response appropriately.

**Author’s response:** Thank you for your feedback.

7. PLOS authors have the option to publish the peer review history of their article ([what does this mean?](https://c05y1x9s.r.us-east-2.awstrack.me/L0/https:%2F%2Fjournals.plos.org%2Fplosone%2Fs%2Feditorial-and-peer-review-process%23loc-peer-review-history/1/010f01926da3ee31-e1098fc4-9bf6-478d-ac73-f4e42a9e89c3-000000/m_89clFTUT44PjXSll4rhuJCeRk=179)). If published, this will include your full peer review and any attached files.


If you choose “no”, your identity will remain anonymous but your review may still be made public.


Do you want your identity to be public for this peer review? For information about this choice, including consent withdrawal, please see our [Privacy Policy](https://c05y1x9s.r.us-east-2.awstrack.me/L0/https:%2F%2Fwww.plos.org%2Fprivacy-policy/1/010f01926da3ee31-e1098fc4-9bf6-478d-ac73-f4e42a9e89c3-000000/qfcG0fljtZzLqvYGFSeWkwmP1x0=179).

Reviewer #1: No

Reviewer #2: No

**Author’s response:** Thank you for your feedback.
